# Study of the Association Between SNPs and External Pelvimetry Measurements in Romanian Simmental Cattle

**DOI:** 10.3390/ani15111586

**Published:** 2025-05-29

**Authors:** Ioana-Irina Spătaru, Alexandru Eugeniu Mizeranschi, Daniela Elena Ilie, Iuliu Torda, Daniel George Bratu, Bianca Cornelia Lungu, Ioan Huțu, Călin Mircu

**Affiliations:** 1 “Horia Cernescu” Research Unit, Faculty of Veterinary Medicine, University of Life Sciences “Regele Mihai I” from Timisoara, 300645 Timisoara, Romania; ioana.spataru@usvt.ro (I.-I.S.); iuliu.torda@usvt.ro (I.T.); daniel.bratu@usvt.ro (D.G.B.); bianca.lungu@fmvt.ro (B.C.L.); calinmircu@usvt.ro (C.M.); 2 Research and Development Station for Bovine Arad, 310059 Arad, Romania; daniela.ilie@scdcbarad.ro; 3 Institute for Advanced Environmental Research, West University of Timisoara, 300223 Timisoara, Romania

**Keywords:** SNPs, external pelvimetry, reproductive traits, Romanian Simmental cattle

## Abstract

The evaluation of external pelvimetry measurements and the genetic factors influencing them is essential for improving morphological characteristics and reproductive performance in cattle. This study represents the first comprehensive analysis of the association between single nucleotide polymorphisms (SNPs) and external pelvimetry traits in Romanian Simmental cattle, a breed recognized for its distinctive pelvic morphology. The relationship between single-nucleotide polymorphisms (SNPs) and external pelvimetry traits—including croup height (CH), buttock height (BH), croup width (CW), rump angle (RA), and croup length (CL)—was examined in Simmental cows. From an initial set of 110 SNPs, 33 markers were retained after applying quality control filters, including a minor allele frequency (MAF) greater than 0.05 and Hardy–Weinberg equilibrium. These SNPs, located on multiple chromosomes, were identified within intronic, exonic, or regulatory regions of relevant genes such as *CLSTN2*, *DPYD*, *FBXL7*, *FBXL13*, *SEMA6A*, *RUNX2*, *FSTL4*, *DST*, *DCBLD2*, *FRMD6*, *CAV2.3*, *ABL2*, *SH3BP4*, *RSBN1L*,and *SAMD12*, suggesting that these genetic variants may influence the development and morphology of the pelvic bones. Statistical analysis revealed significant relationships between certain allele variants and croup measurements, highlighting that the presence of alternative alleles can modify their morphological traits. Notably, the G allele in CLSTN2 reduced croup height by 5.74 cm (*p* = 0.0227), while the T allele in RUNX2 decreased rump angle by 4.49° (*p* = 0.0119).

## Simple Summary

This study explores how single-nucleotide polymorphisms (SNPs) are associated with pelvic conformation in Romanian Simmental cows (alias Romanian Red Spotted Cattle). By examining 33 SNPs located within or in functional regions of important genes, the study aims to understand how these genetic variations affect the shape and size of the pelvic bones, including traits such as croup height (CH), buttock height (BH), croup width (CW), rump angle (RA) and croup length (CL). The results prove that specific genetic variants may influence these traits, which could help improve selection strategies for better reproductive performance in cattle.

## 1. Introduction

Single-nucleotide polymorphisms (SNPs) represent the simplest form of genetic variation between individuals. These variations can be classified as transitions or transversions and occur in the genome at a frequency of approximately one in every 1000 base pairs. SNPs play a significant role in the diversity observed between individuals and in genome evolution. They can affect promoter activity, impacting gene expression, the stability of messenger RNA (mRNA) conformations, and the subcellular localization of RNA and/or proteins, potentially contributing to the onset of various disorders [1]. SNP genotyping technologies are important in breeding programs because they allow the selection of the best animals based on genetic information. In addition, detailed genetic maps based on the use of SNPs are useful for understanding the genetic variations associated with a specific phenotype. Genome-wide association studies (GWAS) represent an essential method for identifying genetic variants associated with complex traits in different cattle breeds [2,3].

This study uses an innovative design by integrating advanced genotyping technologies, focusing on identifying SNPs associated with external pelvimetry traits in a large population of Romanian Simmental cattle [4]. A central reason for this research is the impact of pelvic conformation on fertility and reproductive performance, particularly on calving ease, calf survival at birth, and cow recovery after calving. External pelvimetry traits are known as factors that influence the size of the birth canal and, subsequently, calving difficulty. Neamț et al. (2019) reported that Romanian Simmental cattle experiencing dystocia had significantly smaller pelvic dimensions, particularly narrower ischial width, compared to cows with eutocic calvings (*p* ≤ 0.05), highlighting the critical role of pelvic morphology in calving outcomes and calf viability [5]. Furthermore, a systematic review by Abdela et al. (2016) emphasized that maternal pelvic abnormalities, including reduced pelvic size, are major contributors to dystocia across dairy cattle breeds, negatively affecting calf viability, cow fertility, milk production, and farm profitability [6].

The selection of the 48 candidate genes was based on functional annotation and literature evidence regarding their involvement in skeletal development, bone morphology, and body structure traits in cattle. Although not all genes have been directly associated with pelvic traits through GWAS or QTL studies, several candidates, such as CLSTN2, RUNX2, DPYD, SEMA6A, FBXL7, and SAMD12, have known roles in musculoskeletal development, body growth, and bone structure, justifying their inclusion for further investigation [7,8,9,10,11]. Romanian Simmental cattle were chosen for this study due to their unique skeletal morphology and importance in beef production. While other cattle breeds, such as Holstein, are more commonly studied for traits related to calving ease, Romanian Simmental cattle have distinct pelvic traits that may be influenced by different genetic factors. Additionally, frequency differences in alleles between breeds may lead to varying SNP effects on traits like croup height, rump angle, and body structure. Understanding these breed-specific genetic differences will contribute to the development of more tailored breeding programs for improving reproductive traits in Romanian Simmental cattle.

In this study, we aimed to highlight the relationship between genetic variability and pelvic conformation in Romanian Simmental cattle. Using an association study on genes previously identified as being involved in pelvic conformation, we focused on identifying genetic variants associated with relevant pelvic traits, such as croup width, length, tilt, and rump height, which directly impact the size of the birth canal and, consequently, calving difficulty.

## 2. Materials and Methods

### 2.1. Animals and Phenotypic Records

During 2023–2024, 152 Simmental cattle were measured for external pelvimetry and selected based on the availability of reproductive history and genetic assessments at the Research and Development Station for Bovine—Arad, Romania. All cattle involved in the study were kept in the same housing and feeding conditions and were included in the Official Recording of Milk Production in Romania.

In this study, we initially evaluated 200 Simmental cattle for five external pelvimetry traits. To obtain phenotypic values representing essential points of external pelvimetry (croup height, buttock height, croup length, croup width, and rump angle), we used the Martin pelvimeter, a zoometric rod, and a laser level. The value of the croup height was determined by measuring the distance between the dorsal part of the croup and the floor. For buttock height, the distance between the ischial tuberosity and the floor was measured. The croup length was measured from the iliac tuberosity to the ischial tuberosity. The croup width was measured as the distance between the right and left iliac tuberosities. The rump angle was determined by measuring the angle between the coxal tuberosity and the ischial tuberosity. The laser level shows an angle that indicates the croup’s inclination. If the croup is completely horizontal, the angle will be 0°. If the croup is inclined upwards, the angle will be positive, and if it is inclined downwards, the angle will be negative. All measurements were conducted by a single accredited and trained operator using standardized procedures in accordance with the ICAR Guidelines for Conformation Recording Methods, ensuring consistency and minimizing inter-operator variability in phenotypic data collection. To ensure data quality and consistency, we applied the interquartile range (IQR) method to identify and exclude outliers—specifically, any values falling below Q1 − 1.5 × IQR or above Q3 + 1.5 × IQR. Following this quality control process, 152 animals remained in the final dataset, each with complete measurements across all five traits. Each of these animals had complete records for all five traits, yielding a total of 760 phenotypic observations.

### 2.2. Sampling and Genotyping

Biological samples needed for genotyping consisted of whole blood collected by trained veterinarians. The blood samples (*n* = 152) were extracted from the tail vein in vacutainers containing K3EDTA as an anticoagulant. After collection, the samples were stored in the refrigerator at 4 °C prior to sending them to the IFN Schönow GmbH (Bernaubei, Berlin, Germany) for DNA extraction and genotyping using an Axiom Bovine v3 microarray (Thermo Fisher Scientific, Waltham, MA, USA). The used microarray was based on the reference genome Bos_taurus_UMD_3.1.1. Next [12], the data quality control step was performed with the aim of removing all SNPs (call rates < 95%) and animals (call rates < 95%) with insufficient genotyping quality. SNPs with minor allele frequency (MAF) < 0.05 and those with genotypes not in accordance with the Hardy–Weinberg equilibrium were eliminated using Plink v1.90b6.21 [13], and the remaining missing genotypes were imputed with BEAGLE v. 5.2 [14].

### 2.3. Investigated Genes and SNPs

The SNPs analyzed in this study were selected based on their location within or near candidate genes previously reported in GWAS or QTL studies to be involved in skeletal development, body conformation, or musculoskeletal traits. These genes were considered biologically relevant for exploring potential associations with external pelvic morphology. From the initial set of 110 SNPs located within or near 48 candidate genes mentioned by Gonzalez- Guzman- and Lazaro (2020), Zhang M (2024), Jourshari (2023), Abdalla (2023), Yu (2023), Silva (2024), Lu (2021), Bouwman and Daetwyler (2018), Takasuga (2016), Lin (2022), Van vanhossou (2020); Kim Young-Im (2023), Komori (2024) and Zhang and Sheng (2023) [7,8,9,10,11,15,16,17,18,19,20,21,22,23], only 33 SNPs associated with external pelvimetry traits were retained after applying quality control and statistical significance filters. These SNPs overlapped with 15 genes, which were therefore selected for detailed statistical analysis and interpretation. Detailed functional annotation (exonic, intronic, or intergenic categorization) was provided only for the SNPs that exhibited significant or biologically relevant associations. The remaining non-significant SNPs were not prioritized for in-depth functional characterization, as they fell outside the scope of the final analysis.

Details regarding the SNP probe set ID, gene symbol, chromosome position based on the UMD_3.1.1genome assembly of *Bos Taurus* [12], alleles, and SNP rsID are presented in Table S1 (Supplementary Materials).

### 2.4. Association Analysis

Statistical association analyses were performed using the R programming language v. 4.3.3 23. Descriptive statistics were collected using the psych package v. 2.4.1224 [24,25]. In this study, we employed general linear models (GLMs) to assess the association between single-nucleotide polymorphisms (SNPs) and phenotypic traits. The model was structured as follows:

Y_i_ = μ + SNP_i_ + PC_1_ + PC_2_ + PC_3_ + PC_4_ + PC_5_ + ε_i_, (1)

where Y_i_ represents the phenotypic value for individual i, SNP_i_ denotes the genotype effect coded additively (0, 1, or 2), PC_1_ through PC_5_ are the first five principal components accounting for population structure, and ε_i_ is the residual error term.

**Figure 1:**
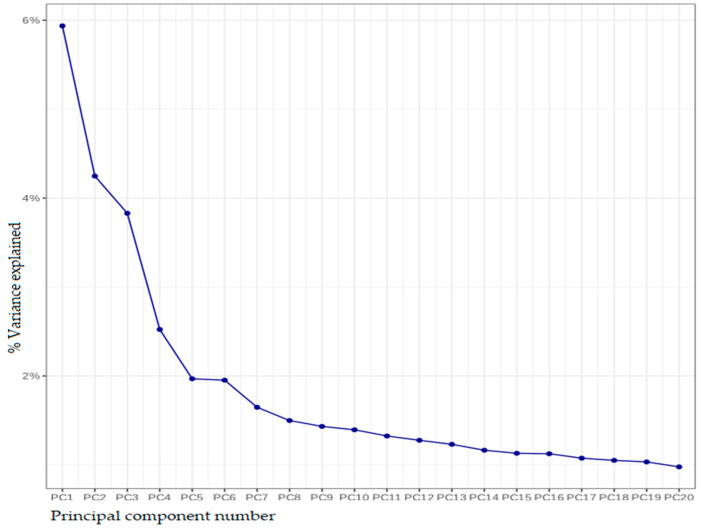
Scree plot of population stratification based on principal component analysis.

Each SNP was tested independently for its association with each trait using this model. Association tests were performed via linear regression models with each pelvimetric trait as a dependent variable, the genotypes as independent variables, and the first 5 principal components included as covariates, to account for population structure effects (Figure 1). Results were deemed statistically significant at a *p*-value threshold of 0.05.

## 3. Results

### 3.1. Value and Variability of Pelvic Measurements in Simmental Cattle

For the females in this population of Simmental cattle, the following results were obtained for the external pelvimetric measurements: the average croup height was 143.73 cm ± 0.30, with an almost symmetric distribution and moderate variability (Table 1). The average buttock height was 126.47 cm ± 0.31, while the croup length was 54.25 cm ± 0.16, with all these traits showing small to moderate variability. The average croup inclination was −0.24° ± 0.18, indicating a slightly inclined croup, and the croup width was 56.68 cm ± 0.19.

### 3.2. Analysis of SNPs Associated with Croup Height (CH)

Based on the statistical analyses described in Equation (1) from Section 2.4, a total of seven SNPs significantly associated with CH were identified in Simmental cattle. The identified genetic markers are located on chromosomes 1, 3, 4, 7, and 14, suggesting that they may influence the morphological development of the pelvis.

On chromosome 1, two SNPs were identified in relation to the CLSTN2 gene: AX-106723587 was classified as an intronic variant, while AX-106750655 was located downstream of the gene. For the SNP AX-106723587, the reference allele is T, and the alternative allele is *G*. When comparing homozygotes (*TT*) with heterozygotes (*TG*), a decrease in CH was observed with an estimated value of −5.74 (*p* = 0.0227) (Table 2). Additionally, the difference observed between homozygotes with the TT genotype and those with the *GG* genotype was −5.33 (*p* = 0.0468).

A third SNP located on chromosome 1, AX-106750655, shows a positive effect on CH, with an estimated value of +1.83 (*p* = 0.0269). This suggests that heterozygotes (*TC*) may exhibit a greater CH compared to homozygotes (*CC*).

On chromosome 4, the SNP AX-106763743 was identified as an intronic variant in the *FBXL13* gene. This SNP is associated (+1.52 (*p* = 0.0489) with CH, suggesting a possible role for the *FBXL13* gene in the growth and development of pelvic bones.

The SNP AX-106753436, classified as an intergenic variant on chromosome 7 in relation to the SEMA6A gene, is associated (−3.82) with croup height (CH), indicating a negative association between the alternative allele (A) and CH, and the *p*-value of 0.0487 confirms the statistical significance of this result.

The results for SNP AX-106724218, located in the intronic region of the *SAMD12* gene, suggest a clear association between the presence of the *T* allele and a decrease in CH. Homozygous individuals for the alternative allele (*TT*) exhibited a significant reduction in this phenotype (−3.87, *p* = 0.0031) compared to homozygous individuals for the reference allele (*CC*).

For SNP AX-117082755, classified as an intronic variant in the *SAMD12* gene, the analysis confirms the influence of the *T* allele on this phenotype. The estimated difference between individuals with the *CC* and *TT* genotypes was −3.31 (*p* = 0.0060), highlighting a similar trend of reduction in CH.

### 3.3. Analysis of SNPs Associated with Buttock Height (BH)

In the analysis, 6 SNPs associated with BH were identified, located on chromosomes 3, 4, 14, and 20.

SNP AX-106733516, located on chromosome 3, classified as an intergenic variant in relation to the *SH3BP4* gene, shows a significant association with BH. The estimated value recorded (−3.07, *p* = 0.0389) suggests that the presence of the *G* allele contributes to a decrease in BH compared to the *T* allele (Table 3).

Analyzing the *AA* and *GG* genotypes for SNP AX-106736322, located in an intron of the *FBXL13* gene, it can be observed that the recorded estimated value of −2.70 indicates a decrease in BH. This result suggests that the presence of the *G* allele contributes to a reduction in BH compared to the *A* allele, as confirmed by the *p*-value of 0.0148. For SNP AX-124375871, located in an intron of the *RSBN1L* gene, the estimated value of +2.17 (*p* = 0.0471) indicates an increase in BH. On chromosome 20, SNP AX-106731384 was identified, located as an intergenic variant in relation to the *FBXL7* gene. Regarding the difference between the *GA* and *AA* genotypes, the positive estimated value (+2.23) indicates an increase in BH. The *A* allele is involved in determining the increase in BH, with a more pronounced effect in homozygotes (*AA*) compared to heterozygotes (*GA*).

### 3.4. Analysis of SNPs Associated with Rump Angle (RA)

The statistical genomic analysis highlighted the presence of 7 SNPs located on chromosomes 3, 7, 16, and 23, which were subsequently examined for their potential implications regarding RA.

On chromosome 3, three SNPs associated with significant changes in RA were identified. Depending on the genetic variant present, these SNPs were linked to both positive and negative effects on the analyzed phenotype. For SNP AX-106751591, the difference between the heterozygous genotype (*AG*) and the homozygous genotype (*GG*) is reflected by the positive estimated value (+1.06), suggesting an association between the *G* allele and an increase in RA. This is further confirmed by the *p*-value of 0.0393, which is below the significance threshold (Table 4).

When comparing the two homozygous genotypes (*CC* and *TT*) for SNP AX-106735685, it can be observed that the positive estimated value (+1.89) indicates an increase in RA. The alternative *T* allele is associated with this increase, and the *p*-value of 0.0263 confirms the statistical significance of the relationship between the analyzed variables.

For SNP AX-124348371, the estimated value (+2.07) highlights the significant comparison (*p* = 0.0130) between the homozygous genotypes *GG* and *TT*, suggesting an association of the alternative *T* allele with an increase in RA. These three SNPs are located in relation to the DPYD gene, with one classified as an intergenic variant (AX-106751591) and the other two as intronic variants (AX-106735685 and AX-124348371), suggesting a role for this gene in the growth and development of pelvic bones, including influencing RA.

On chromosome 7, SNP AX-106727722 was identified in an intergenic region close to the *FSTL4* gene. Analyzing the homozygous genotypes *TT* and *CC*, the estimated value of −1.57 suggests that the alternative *C* allele is associated with a decrease in RA.

Regarding chromosome 16, SNPs AX-106724034 and AX-106742186 were identified.SNP AX-106724034 is located as an intergenic variant in relation to the *CAV2.3* gene and presents the reference allele *T* and the alternative allele *C*. Analyzing individuals with homozygous genotypes (*TT*) and heterozygous genotypes (*TC*), a decrease in RA was observed with the estimated value of −1.73 (*p* = 0.0247). In the case of SNP AX-106742186, located in an intergenic region close to the *ABL2* gene, the *G* allele is associated with a significant increase in RA compared to the *A* allele. Analyzing the two genotypes, it was found that homozygotes (*GG*) exhibit a greater RA than heterozygotes (*GA*), with the estimated value being +1.36.

SNP AX-124384326, located within an intergenic region adjacent to the *RUNX2* gene, is associated with RA in the cattle in this study. When comparing the homozygous genotypes *CC* and *TT*, the estimated value (−3.89, *p* = 0.0330) indicates that the alternative *T* allele is associated with a decrease in RA.

### 3.5. Analysis of SNPs Associated with Croup Length (CL)

Three SNPs significantly associated with this trait have been identified on chromosomes 16 and 23.

SNP AX-106752137, located in an intron of the CAV 2.3 gene on chromosome 16, showed a significant association with CL. Comparing the *TT* and *TC* genotypes, it can be observed that the positive estimated value (2.12, *p* = 0.0105) indicates an increase in CL. Additionally, the comparison between the homozygous *TT* and *CC* genotypes yields an estimated value of 2.26 and a *p*-value of 0.0215, suggesting that the *C* allele is associated with a more pronounced increase in CL (Table 5).

Regarding SNP AX-117088037, located in an intergenic region near the *DST* gene, the positive estimated value (1.41) indicates a significant association between this genetic variant and croup length. The observed difference between the *CC* and CT genotypes suggests that the T allele may be associated with an increase in croup length. Additionally, the *p*-value of 0.0375, which falls below the 0.05 threshold, supports the hypothesis of a significant association between this SNP and croup length.

### 3.6. Analysis of SNPs Associated with Croup Width (CW)

In our analysis, 5 SNPs located on chromosomes 1, 10, and 16 were identified and subsequently significantly associated with croup width. On chromosome 1, two SNPs were identified in relation to the *DCBLD2* gene (AX-106742670: intergenic; AX-115103182: intronic). Analyzing the *TC* and *CC* genotypes for SNP AX-106742670, it was found that the alternative *C* allele is associated with an increase in CW. This association is supported by the estimated effect value of 1.19 and a *p*-value of 0.0236, indicating a statistically significant association. On the other hand, for the *CT* and *TT* genotypes for SNP AX-115103182, it was observed that the *T* allele is associated with a decrease in CW. This is reflected by the estimated value of −1.27 and a *p*-value of 0.0318 (Table 6).

When comparing the two homozygous genotypes *CC* and *GG* for SNP AX-106763243, it was observed that the positive estimated value (1.53, *p*-value = 0.0320) indicates a significant difference between them. The presence of the *G* allele is associated with an increase in CW, highlighting its significant effect on the development of the trait.

For SNP AX-124386523, the heterozygous genotype (*GA*) exhibits a smaller CW than the homozygous genotype (*GG*), and the negative estimated value (−1.63, *p* = 0.0157) confirms this aspect. This reflects the fact that the alternative allele A is associated with a decrease in CW.

SNP AX-106724034, positioned within an intergenic region on chromosome 16, in proximity to the *CAV2.3* gene, shows an estimated value of 2.16 and a *p*-value of 0.0050, indicating a significant influence on CW. Analyzing the *TT* and *TC* genotypes, it was found that heterozygotes show a smaller CW than homozygotes, emphasizing the effect of the C allele on the analyzed phenotype.

## 4. Discussion

In this exploratory study, we did not apply a Bonferroni correction due to the moderate sample size (*n* = 152) and the relatively small number of SNPs analyzed (33 SNPs). Instead, we considered associations significant at a nominal *p*-value threshold of *p* < 0.05. However, as no correction for multiple testing was applied, some of the identified associations may be false positives. The following genes and SNPs were associated with our phenotype measurements.

The *CLSTN2* gene (Calsyntenin 2) is involved in lipid metabolism and plays an important role in the proliferation of adipocytes in both visceral and subcutaneous adipose tissue. The expression of the *CLSTN2* gene is associated with glucose and insulin metabolism, contributing to their regulation and the onset of metabolic disorders. Fluctuations in insulin and glucose levels can influence the regulation of the endocrine axis, directly impacting reproductive processes and the onset of sexual maturity [26,27]. It has been previously reported that the *CLSTN2* gene plays a role in influencing hip width [9]. We found that in the case of SNP AX-106723587, located in an intron of the *CLSTN2* gene, the presence of the *G* allele is associated with a reduction in CH. Our findings suggest that although the *CLSTN2* gene does not have a direct effect on CH, the proximity of these three SNPs suggests a possible indirect influence on the trait through pleiotropic mechanisms. It is possible that the significant SNPs identified are in linkage disequilibrium with causal variants not directly genotyped. Further LD block estimation and fine-mapping will be required to refine these associations.

The *DPYD* gene (Dihydropyrimidine Dehydrogenase) encodes the enzyme dihydropyrimidine dehydrogenase, which is responsible for the degradation of pyrimidines, particularly uracil and thymine, when they are no longer needed. The enzyme initiates the pyrimidine degradation process by converting uracil into 5,6-dihydrouracil and thymine into 5,6-dihydrothymine. The products resulting from this process are either eliminated from the body or redistributed into other metabolic pathways [28]. In cattle, the *DPYD* gene contributes to maintaining energy balance, supports oxidative metabolism, and participates in the efficient use of nutrients in the body. According to the study conducted by Jourshari (2023), SNPs located near the *DPYD* gene have been associated with variation in hip width in cattle [9]. The authors also suggest that this gene may be involved in meat quality and fatty acid composition in local cattle breeds in China [28]. Our results emphasize the fact that the *G* allele for SNP AX-185119475, located in an intron of the *DPYD* gene, is associated with a reduction in CH, possibly by influencing the growth and development of the bone structures in this region.

In the study reported by Guzman et al. (2020), the *FBXL13* gene was identified in a region associated with the width of the ilium and body length in heifers [7]. The *FBXL13* gene is a protein from the F-box family, characterized by a specific domain of about 40 amino acids. It is integrated into SCF (SKP1-CUL1-F-box) complexes that act as E3 ubiquitin ligases, playing an essential role in ubiquitination and degradation of target proteins [29]. According to our results, the analyzed SNPs, AX-106763743 and AX-106736322, were located in introns of the *FBXL13* gene, which had previously been discussed in the context of CH. Although the genotypes differed in the case of CH, it appeared that SNPs located near the *FBXL13* gene played an important role in the development of pelvic bones [29].

The *SEMA6A* gene, also known as semaphorin 6A, is involved in various essential processes such as cell migration, axon guidance, and synaptogenesis. In studies on mice, mutations in the *SEMA6A* gene have been associated with defects in cell migration and axon guidance in different regions of the brain, including the thalamocortical system, hippocampus, and cerebellum. These defects lead to improper development of neural networks [30]. In the study by Zhang et al. (2024), SNPs located near the *SEMA6A* gene were associated with CL in Xinjiang Brown cattle, explaining approximately 0.093% of the variation in this trait in the context of their research [8]. Our results highlight that the contrast between SNP0 and SNP2 for SNP AX-106753436, located in an intergenic region close to the *SEMA6A* gene, highlights the difference between homozygotes for the reference allele (*CC*) and those for the alternative allele (*AA*), indicating a reduction in CH in individuals carrying the *A* allele.

Genomic analysis highlighted the presence of four SNPs on chromosome 14 associated with this phenotype, all located near the SAMD12 gene. Guzman et al. (2020) identified a genomic region on chromosome 14 that includes the *SAMD12* gene and is associated with CL in Murrah buffaloes [7]. This emphasizes the pleiotropic effect of the *SAMD12* gene on pelvic conformation. The *SAMD12* gene encodes a protein involved in the tyrosine kinase receptor signaling pathway, which is active on the inner surface of the plasma membrane. Zhuang et al. (2020) found a significant association of SNPs located onthis gene with body weight in 18-month-old Simmental cattle [31]. Later, Mancin et al. (2022) confirmed the relevance of this region, identifying a SNP near the *SAMD12* gene associated with carcass traits [32]. In our results, for SNP AX-106724218, heterozygous individuals (*CT*) showed an estimated value of −3.77 (*p* = 0.0019), falling between the values observed in homozygotes, suggesting a progressive effect of the *T* allele on this trait. Similarly, for SNP AX-117082755, heterozygous (*CT*) individuals showed a less pronounced reduction of −2.56 (*p* = 0.0262), indicating that the effect of the *T* allele becomes more evident in homozygous individuals due to the presence of two copies of this allele.

In Nellore cattle, Machado et al. (2022) associated the *SEMA6A* gene with muscle development [33]. The authors suggest that the *SEMA6A* gene is involved in regulating biological processes, including the development and growth of muscle tissue. Furthermore, it plays a role in RNA synthesis, energy metabolism, and response to external stimuli [33]. Given the close connection between muscle and bone structures, the *SEMA6A* gene could indirectly influence CH as well.

The *SH3BP4* gene (SH3 domain binding protein 4) acts as an inhibitor of Rag GTPase activity, an important component of the mTORC1 complex. This protein complex controls essential cellular processes such as cell growth, protein synthesis, and autophagy, responding to nutritional, hormonal, and stress signals. Thus, by controlling the activity of the mTORC1 complex, the *SH3BP4* gene influences the maintenance of energy balance and the adaptation of cells to stress conditions [16,17]. In the study conducted by Lu et al. (2021), the SNP identified near the *SH3BP4* gene was associated with croup slope, suggesting an influence on the development of pelvic bones [16]. Butterfield et al. (2021) identified the SH3BP4 gene as having a functional role in the pathogenesis of osteoarthritis in mice. They found that the absence of the gene led to the early development of osteoarthritis, manifested by the degeneration of articular cartilage. The *SH3BP4* gene is also involved in the regulation of cellular signaling by influencing the processes of transferrin receptor transport and other pathways essential for cellular homeostasis [34]. These processes highlight the role of the *SH3BP4* gene in the proper functioning of bone and cartilage tissues. Furthermore, the way it regulates cell growth and development could contribute to the formation of the pelvic structure, including traits such as BH. Based on statistical analyses and estimated value recorded for SNP AX-106733516, we concluded that homozygotes (*GG*) exhibited a lower value for this trait compared to homozygotes (*TT*).

The *RSBN1L* gene (Round Spermatid Basic Protein 1 Like) encodes a protein involved in spermatogenesis and oogenesis processes. It shares a common origin with the *RSBN1L* gene. Available information about *RSBN1L* highlights its expression in the testis, brain, and ovary. Given the structural and functional similarity between the two genes, these findings may also be relevant for the *RSBN1L* gene [35]. *RSBN1L* is a protein involved in chemical reactions that require oxygen and interacts with metal ions, being active in the nucleus. It also acts as a specific demethylase, removing methyl (-CH3) groups from lysine residues in proteins, which can influence their structure and function [35]. Guzman et al. (2020) identified a SNP in buffaloes located on chromosome 4, on the *RSBN1L* gene, associated with ilium width and body length [7]. The results indicate that SNPs located on the *FBXL13* and *RSBN1L* genes can influence both the BH and other structures that form the pelvis. Our results show that in the case of SNP AX-124375871 in an intron of the *RSBN1L* gene, a positive association was found, which is attributed to the A allele. This allele causes a more pronounced manifestation of BH growth in heterozygotes (*GA*), compared to homozygotes (*GG*).

Rothammer et al. (2013) suggest that the *SAMD12* gene plays an important role in Creole cattle breeds in adapting to environmental conditions and in their performance related to milk and meat production. The authors state that certain alleles of this gene have been selected, suggesting that *SAMD12* could be essential for improving performance in cattle [36]. Regarding the values we have obtained, the *G* allele of SNP AX-117082755 is associated with a decrease in BH, and its effect is more pronounced in heterozygotes (*AG*). These observations suggest that SNPs located near the *SAMD12* gene may influence BH. Additionally, the *T* and *G* alleles of SNP AX-117082755 and SNP AX-124375871have different effects on this phenotype, indicating a possible role of the *SAMD12* gene in determining pelvic structures.

In the study by Abdalla et al. (2021), the SNP located on the *FBXL7* gene was associated with a trait that influences the positioning of the animal’s hind limbs when viewed from behind. This trait is important for mobility because the position of the hind limbs influences the efficiency of movement, and the *FBXL7* gene plays a role in the development and functioning of the musculoskeletal system [10]. The *FBXL7* gene (F-Box and Leucine-Rich Repeat Protein 7) encodes a protein from the F-box family that regulates mitotic cell cycle progression and apoptosis [37]. Wu et al. (2018) identified an SNP located on chromosome 16in the *FBXL7* gene. The authors suggest that the *FBXL7* gene may influence the reproductive performance of sows from the Landrace and Large White breeds, impacting the total number of piglets born, the total number of live piglets, and their total birth weight [38]. The *FBXL7* gene also impacts mitochondrial function and cellular energy stability [37]. Our obtained *p*-value of 0.0098 suggests that this association is statistically significant, indicating a possible role of the *FBXL7* gene in determining BH. Our results align with those obtained by Lu et al. (2021) in Holstein cows in China. Similarly, they identified a SNP on chromosome 7, located near the *FSTL4* gene, which they associated with RA [16]. The *FSTL4* gene (Follistatin-like 4) is a member of the follistatin family and an inhibitor of growth and TGF-β. *FSTL4* is involved in regulating the functions of mesenchymal cells and in the early development of the nervous and ocular systems [39]. In cattle, the *FSTL4* gene is involved in regulating ovarian function, playing a role in ovulation and corpus luteum formation [40]. Dewison et al. (2023) suggest that the *FSTL4* gene is a potential indicator of oocyte quality due to its increased expression in fertilized oocytes that have reached the blastocyst stage [41].We found that the *p*-value of 0.0361 confirms that the association for SNP AX-106727722 is statistically significant.

The *CAV2.3* gene encodes the protein responsible for producing type R calcium channels. These channels are involved in conducting calcium ions into nerve and muscle cells. Calcium channels are also essential for neuronal and muscular excitability, and mutations in the *CAV2.3* gene can be associated with various neurological disorders [42]. SNPs located in this gene suggest a possible influence on the formation of bone structures, including RA, an essential morphological trait that depends on the proper development of the pelvic bones and musculature. Our results are consistent with those observed by Zhang et al. (2024) [8], who associated the SNP located near the *CAV2.3* gene with CL, indicating a similar influence on this trait [18]. In our study, the decrease in RA was recorded in individuals with the *TT* and *CC* genotypes, with an estimated value of −1.63 and a *p*-value of 0.0317, indicating a statistically significant association. These results suggest that the *C* allele might be associated with a reduction in RA.

*ABL2* is a proto-oncogene with tyrosine kinase activity and is not associated with specific membrane receptors. It plays a crucial role in regulating the actin cytoskeleton, influencing cell morphology and motility, as well as cell adhesion to the extracellular matrix. The gene contains a tyrosine kinase domain and two domains that bind to F-actin, which are essential for its functions [43,44]. The *ABL2* gene is involved in the innate immune response, influencing processes such as cell proliferation, migration, and differentiation. It also regulates important metabolic processes, such as food intake in cattle and the thickness of dorsal fat in pigs. These aspects influence the ability of animals to adapt to different food sources and respond to metabolic stress or challenging environmental conditions [45]. Moreover, *ABL2* influences the proliferation and fusion of myoblasts, allowing for the proper development of muscle fibers. In the study conducted by Lee et al. (2017) on mice, it was found that the absence of the *ABL2* gene leads to excessive proliferation and fusion of myoblasts, which can cause changes in the structure and size of muscle fibers [45]. Van vanhossou et al. (2020) identified an SNP located near the *ABL2* gene, which they associated with hip width in Holstein cattle. This suggests that the *ABL2* gene may also influence RA, impacting pelvic conformation [20]. The resulting *p*-value of 0.0288 we have obtained for AX-106742186 confirms the statistical significance of this association, indicating that the *G* allele plays an important role in determining RA.

*RUNX2* is essential not only for osteoblast differentiation but also for chondrocyte maturation [22]. The results previously obtained demonstrate that the influence of the *RUNX2* gene on SNP AX-124384326 could play an important role in modifying the shape of the croup, being a relevant genetic factor in the development of pelvic structure in cattle [8]. Deletion of the *RUNX2* gene impairs the development of both oocytes and spermatozoa and hinders the development of intramembranous and endochondral bones. *RUNX2* expression is present in all cells of the osteoblast lineage, including osteoprogenitors, preosteoblasts, immature osteoblasts, mature osteoblasts, osteocytes, and chondrocytes [21,22]. Based on our results, regarding the contrast between the *CC* and *CT* genotypes, the recorded estimated value (−4.49, *p* = 0.0119) suggests a more pronounced decrease in RA, and the *T* allele continues to be associated with this significant modification.

The *DST* gene encodes the protein dystonin, which is essential for maintaining the structure of the cellular cytoskeleton. It is part of the Plakin family and plays a role in maintaining the stability of the cytoskeleton by facilitating the connection between its different components, such as actin and microtubules. It is also involved in the stability and physiological function of muscular, nervous, and epidermal tissues [46]. Zhang et al. (2024) identified an SNP located on chromosome 23, near the *DST* gene, which they associated with CL in Xinjiang Brown cattle [8]. According to our obtained *p*-value of 0.0375, which is below the 0.05 threshold, supports the hypothesis of a significant association between this SNP and CL.

The *DCBLD2* gene (Discoidin, CUB, and LCCL domain containing 2) is located on the cell surface of the plasma membrane and is involved in cell migration and interaction. It also plays an important role in regulating the activity of signaling receptors, influencing how cells respond to signals from their environment [47]. In the study conducted by Zhang et al. (2024), a significant association was highlighted between an SNP located on chromosome 1 and CL [8]. The authors suggested that the *DCBLD2* gene, located near this SNP, may influence the development of pelvic bones [8]. These results are relevant to our study, providing an example of how SNPs located near certain genes can influence important morphological traits. In our study, we found that depending on the SNP location, the *DCBLD2* gene might increase the CW (when it is close to SNP AX-106742670, depending on the *C* allele) or decrease (when it is located on SNP AX-115103182, influenced by the *T* allele).

SNPs AX-106763243 and AX-124386523, located on chromosome 10, are significantly associated with CW. Both SNPs are near the *FRMD6* gene, highlighting its influence on croup development. Yu et al. (2023) obtained similar results to ours, identifying an SNP located on chromosome 10 near the *FRMD6* gene, which they associated with CL [11]. The *FRMD6* gene (FERM domain containing 6) is located in the cytoplasm and at the plasma membrane. This gene is involved in cellular signaling and regulates cellular senescence processes. FRMD6 stimulates the Hippo signaling pathway, activating the MST kinase and inactivating the YAP/TAZ proteins, which are essential for controlling cellular growth and development [11]. Through the Hippo-YAP-CCN3 signaling axis, the *FRMD6* gene controls the cell fate toward senescence, regulating markers such as p21 and p16. Additionally, *FRMD6* is influenced by the transcription factors p53 and SMAD, which control cellular responses to external signals, including TGF-β [48]. Concerning the SNPs located in introns of the *FRMD6* gene, the alternative allele *A* (on SNP AX-124386523) is associated with a decrease of CW, while the presence of the G allele accompanies an increase of CW, meaning that SNP AX-106763243 might influence the development of the trait.

A limitation of our study is the selective inclusion of SNPs based on their known functional relevance to pelvic conformation traits. While this targeted approach enhances the focus on biologically plausible candidates, it may inadvertently exclude other SNPs that could be associated with the traits of interest. To address this, we recommend that future studies incorporate a broader range of SNPs from the Axiom chip to facilitate more comprehensive association analyses. Our studies highlights the association between single nucleotide polymorphisms (SNPs) and external pelvimetry traits in Romanian Simmental cattle, based on a sample of the same animals previously analyzed in a GWAS for mastitis [49]. We analyzing 33 SNPs across multiple chromosomes to understand their influence on croup height (CH), buttock height (BH), croup width (CW), rump angle (RA), and croup length (CL) [4]. The identified SNP markers associated with external pelvimetry traits, such as croup width, rump angle, and croup height, have direct relevance to breeding goals in Romanian Simmental cattle. Since pelvic conformation strongly influences calving ease, selecting animals carrying favorable alleles for wider and better-angled pelvis structures could help reduce dystocia rates. Moreover, improving calving ease is correlated with enhanced cow longevity, as cows experiencing fewer calving complications are more likely to remain productive for multiple lactations. Therefore, the SNPs identified in this study could be integrated into existing genomic selection indices, reinforcing selection for reproductive efficiency and extended herd life in Romanian Simmental populations. Although the SNPs identified in this study are located in intronic or intergenic regions and do not alter amino acid sequences, such non-coding variants may still have functional consequences. Intronic SNPs can influence splicing efficiency, transcription factor binding, or mRNA stability. Intergenic variants may affect enhancer or silencer elements, thus modulating gene expression at short or long distances. Considering that the associated genes are involved in skeletal or reproductive development, these non-coding SNPs may exert regulatory effects on pelvic morphology. These mechanisms were considered during gene interpretation, assuming that the identified non-coding variants may influence gene expression or regulatory pathways related to skeletal or reproductive development. In this study, genes were retained for interpretation and discussion only if their associated SNPs showed a statistically significant relationship with at least one of the analyzed pelvic traits. Although the associations are statistical in nature, they were considered potentially relevant based on general biological reasoning, including possible implications in growth, reproductive function, metabolism, or skeletal development.

## 5. Conclusions

This study focuses on five croup traits such as: croup height (CH), buttock height (BH), croup width (CW), rump angle (RA), and croup length (CL), which were selected as parameters of reproductive capacity in Simmental cattle.

The identified SNPs are associated with the measurements involved in external pelvimetry. A total of 33 SNPs were identified that affect the development and morphology of the pelvic bones. The found genetic markers are located on chromosomes 1, 3, 4, 7, 10, 14, 16, and 23, suggesting that they may influence the morphological development of the pelvis. We also found some genes associated with CH, BH, CW, RA, and CL that play an important role in these traits in cattle. In certain situations, the proximity of SNPs suggests a possible indirect influence on the trait through pleiotropic mechanisms.

The diversity in the localization of the SNPs highlights the complexity of the genetics of pelvic traits and provides the possibility of interactions between different chromosomal regions that contribute to the development of pelvic traits. These results can be applied in genetic selection programs to improve the pelvic conformation of cattle, while also reducing the risks associated with calving difficulties and enhancing reproductive performance.

Further investigation and research of these SNPs is necessary to better understand their role in the development of the pelvis. Additionally, future research could explore how these genetic markers interact with other genetic and environmental factors that influence pelvic traits.

## Figures and Tables

**Table 1:** Descriptive analysis of external pelvimetry measurements (*n* = 152).

Variable (ACRONYM) (Measure Units)	Mean	SD	Median	Min	Max	Skewness	Kurtosis	SE
Croup height (CH) (cm)	143.73	3.73	144	134	152.0	−0.28	−0.46	0.30
Buttock height (BH) (cm)	126.47	3.80	126	117	137.0	−0.01	−0.08	0.31
Rump angle (RA) (°)	−0.24	2.17	0	−6	5.5	0.09	−0.23	0.18
Croup length (CL) (cm)	54.25	1.92	54	50	58.0	−0.07	−0.63	0.16
Croup width (CW) (cm)	56.68	2.35	57	50	62.0	−0.18	0.02	0.19

Legend: *n*—number of cases; SD—standard deviation; Skewness—measures the asymmetry of the data distribution. If the skewness is positive, the distribution has a long right tail (positive skew); if it’s negative, the distribution has a long left tail (negative skew); Kurtosis—measures the “tailedness” of the data distribution; high kurtosis indicates heavy tails and more outliers, while low kurtosis suggests a distribution with lighter tails; SE—standard error.

**Table 2:** SNP localization on chromosomes and allele effects on croup height (CH).

SNP	Chr	Reference Allele	Alternative Allele	Contrast	Estimated Value	*p*-Value	Gene
AX-106723587	1	*T*	*G*	SNP0–SNP1	−5.74	0.0227	*CLSTN2*
AX-106723587	1	*T*	*G*	SNP0–SNP2	−5.33	0.0467	*CLSTN2*
AX-106750655	1	*T*	*C*	SNP1–SNP2	1.83	0.0269	*CLSTN2*
AX-185119475	3	*A*	*G*	SNP0–SNP1	−2.86	0.0094	*DPYD*
AX-106763743	4	*C*	*T*	SNP1–SNP2	1.51	0.0489	*FBXL13*
AX-106753436	7	*C*	*A*	SNP0–SNP2	−3.82	0.0487	*SEMA6A*
AX-106724218	14	*C*	*T*	SNP0–SNP2	−3.87	0.0031	*SAMD12*
AX-106724218	14	*C*	*T*	SNP0–SNP1	−3.77	0.0019	*SAMD12*
AX-117082755	14	*C*	*T*	SNP0–SNP1	−2.56	0.0261	*SAMD12*
AX-117082755	14	*C*	*T*	SNP0–SNP2	−3.31	0.0060	*SAMD12*

**Table 3:** SNP localization on chromosomes and allele effects on buttock height (BH).

SNP	Chr	Reference Allele	Alternative Allele	Contrast	Estimated Value	*p*-Value	Gene
AX-106733516	3	*T*	*G*	SNP0–SNP2	−3.07	0.0389	*SH3BP4*
AX-106736322	4	*A*	*G*	SNP0–SNP2	−2.70	0.0148	*FBXL13*
AX-124375871	4	*G*	*A*	SNP0–SNP1	−2.17	0.0471	*RSBN1L*
AX-117082755	14	*C*	*T*	SNP1–SNP2	−1.77	0.0266	*SAMD12*
AX-185112171	14	*A*	*G*	SNP0–SNP1	2.25	0.0326	*SAMD12*
AX-106731384	20	*G*	*A*	SNP1–SNP2	2.23	0.0098	*FBXL7*

**Table 4:** SNP localization on chromosomes and allele effects on rump angle (RA).

SNP	Chr	Reference Allele	Alternative Allele	Contrast	Estimated Value	*p*-Value	Gene
AX-106751591	3	*A*	*G*	SNP1–SNP2	1.06	0.0393	*DPYD*
AX-106735685	3	*C*	*T*	SNP0–SNP2	1.89	0.0263	*DPYD*
AX-124348371	3	*G*	*T*	SNP0–SNP2	2.07	0.0130	*DPYD*
AX-106727722	7	*T*	*C*	SNP0–SNP2	−1.57	0.0361	*FSTL4*
AX-106742186	16	*G*	*A*	SNP0–SNP1	1.36	0.0288	*ABL2*
AX-106724034	16	*T*	*C*	SNP0–SNP2	−1.73	0.0247	*CAV2.3*
AX-106724034	16	*T*	*C*	SNP0–SNP1	−1.63	0.0317	*CAV2.3*
AX-124384326	23	*C*	*T*	SNP0–SNP2	−3.89	0.0330	*RUNX2*
AX-124384326	23	*C*	*T*	SNP0–SNP1	−4.49	0.0119	*RUNX2*

**Table 5:** SNP localization on chromosomes and allele effects on croup length (CL).

SNP	Chr	Reference Allele	Alternative Allele	Contrast	Estimated Value	*p*-Value	Gene
AX-106752137	16	*T*	*C*	SNP0–SNP1	2.12	0.0105	*CAV2.3*
AX-106752137	16	*T*	*C*	SNP0–SNP2	2.26	0.0215	*CAV2.3*
AX-117088037	23	*C*	*T*	SNP0–SNP1	1.41	0.0375	*DST*

**Table 6:** SNP localization on chromosomes and allele effects on croup width (CW).

SNP	Chr	Reference Allele	Alternative Allele	Contrast	Estimated Value	*p*-Value	Gene
AX-106742670	1	*T*	*C*	SNP1–SNP2	1.19	0.0236	*DCBLD2*
AX-115103182	1	*C*	*T*	SNP1–SNP2	−1.27	0.0318	*DCBLD2*
AX-106763243	10	*C*	*G*	SNP0–SNP2	1.53	0.0320	*FRMD6*
AX-124386523	10	*G*	*A*	SNP0–SNP1	−1.63	0.0157	*FRMD6*
AX-106724034	16	*T*	*C*	SNP0–SNP1	2.16	0.0050	*CA_V_2.3*

## Data Availability

No new data were created or analyzed in this study. Data sharing is not applicable to this article.

## Supplementary Materials

The following supporting information can be downloaded at: https://www.mdpi.com/article/10.3390/ani15111586/s1, Table S1: Details of genes, chromosome location, and genomic location of the studied SNPs.

## Abbreviations

BH	Buttock height
CH	Croup height
Chr	Chromosome
CL	Croup length
CW	Croup width
F-actin	Filamentous actin
GWAS	Genome-wide association studies
RA	Rump angle
SMAD	Suppressor of Mothers against Decapentaplegic
SNP	Single nucleotide polymorphism
TGF-β	Transforming growth factor β
