# Supplementary material for: Study of the Association Between SNPs and External Pelvimetry Measurements in Romanian Simmental Cattle
Source: Animals (Basel). 2025 May 29;15(11):1586. doi: 10.3390/ani15111586 (PMC12153695; doi:10.3390/ani15111586)
Supplement: Supplementary file 1 [file animals-15-01586-s001.zip › animals-3594106-supplementary.pdf]

Table S1. Details of genes, chromosome location and genomic location of the studied SNPs.

SNP Probe Set ID	Gene symbol	Chr	Position[1]	Allele A	Allele B	SNP rsID
AX-106734868	*MYH15*	1	53551656	T	C	-
AX-106732173	*MYH15*	1	53582877	A	G	-
AX-106720648	*MYH15*	1	53630189	T	G	-
AX-106750397	*MYH15*	1	53656600	A	G	-
AX-124378375	*MYH15*	1	53679183	T	C	-
AX-106735753	*DCBLD2*	1	42749580	T	C	-
AX-106742670	*DCBLD2*	1	42771916	T	C	-
AX-115103182	*DCBLD2*	1	42799445	T	C	rs43229755
AX-106750655	*CLSTN2*	1	129388513	T	C	rs110780956
AX-106723587	*CLSTN2*	1	129424205	T	G	rs43263856
AX-106756021	*CLSTN2*	1	129505485	T	C	-
AX-106723593	*ATG4C*	3	83306806	T	G	-
AX-106733516	*SH3BP4*	3	115210793	T	G	-
AX-106751591	*DPYD*	3	45826189	A	G	-
AX-185119475	*DPYD*	3	45880538	A	G	rs378500362
AX-106734473	*DPYD*	3	45996935	T	C	rs43712282
AX-124348371	*DPYD*	3	46086314	T	G	rs109495108
AX-106735685	*DPYD*	3	46134907	T	C	rs110776492
AX-106762083	*DPYD*	3	46318628	A	C	rs109580467
AX-185119477	*DPYD*	3	46405331	A	G	rs378406199
AX-106762436	*DPYD*	3	46410484	A	G	rs110001765
AX-115104470	*PTPN12*	4	43860090	T	G	-
AX-106733848	*RSBN1L*	4	43710013	T	C	rs110163880
AX-124375871	*RSBN1L*	4	43763107	A	G	rs29025758
AX-106747158	*CCDC146*	4	44177870	A	G	-
AX-106757570	*FAM185A*	4	44359029	T	C	-
AX-171444094	*FBXL13*	4	44406478	T	C	rs108967918
AX-106736322	*FBXL13*	4	44469848	A	G	rs43388331
AX-106729600	*FBXL13*	4	44505761	T	C	-
AX-106763743	*FBXL13*	4	44542142	T	C	rs42765191
AX-106729600	*LRRC17*	4	44505761	T	C	-
AX-115104470	*PTPN12*	4	43860090	T	G	-
AX-106747158	*CCDC146*	4	44177870	A	G	-
AX-106724406	*ARMC10*	4	44612810	A	C	rs42767622
AX-106754981	*NAPEPLD*	4	44671762	A	G	-
AX-106721536	*NAPEPLD*	4	44694369	A	G	-
AX-106757570	*FAM185A*	4	44359029	T	C	-
AX-106733848	*RSBN1L*	4	43710013	T	C	rs110163880
AX-124375871	*RSBN1L*	4	43763107	A	G	rs29025758

SNP Probe Set ID	Gene symbol	Chr	Position[1]	Allele A	Allele B	SNP rsID
AX-171444094	*FBXL13*	4	44406478	T	C	rs108967918
AX-106736322	*FBXL13*	4	44469848	A	G	rs43388331
AX-106729600	*FBXL13*	4	44505761	T	C	-
AX-106763743	*FBXL13*	4	44542142	T	C	rs42765191
AX-106729600	*LRRC17*	4	44505761	T	C	-
AX-106725313	*COL1A2*	4	11649948	A	G	rs110913617
AX-106747250	*CCND2*	5	106269362	T	C	rs110421124
AX-106754519	*DMP1*	6	104305915	A	C	-
AX-115113278	*LCORL*	6	38845992	A	C	rs110961068
AX-106731669	*LCORL*	6	38869785	T	C	rs109294917
AX-106754365	*CHSY3*	7	25210041	A	G	rs41592021
AX-106734626	*CHSY3*	7	25420525	C	G	rs41657989
AX-106753436	*SEMA6A*	7	38780609	A	C	-
AX-106727722	*FSTL4*	7	46477516	T	C	-
AX-185104904	*FSTL4*	7	46493107	A	G	rs382678897
AX-106722191	*EPHX2*	8	75919385	A	G	rs43436859
AX-106749022	*STMN4*	8	75650021	A	G	-
AX-106758983	*CHRNA2*	8	75874902	A	G	rs110370186
AX-106738750	*SMU1*	8	76152259	T	C	rs110061505
AX-106721012	*GULO*	8	76005495	A	G	-
AX-169380681	*RFX6*	9	34163790	A	G	rs135889862
AX-106741677	*RFX6*	9	34176604	A	G	rs109252847
AX-115106312	*KPNA5*	9	34317516	A	G	-
AX-124377038	*NEPN*	9	33618865	A	G	rs41656796
AX-185123100	*ROS1*	9	33818808	A	G	rs379828862
AX-117083515	*FRMD6*	10	44362494	T	C	-
AX-185110014	*FRMD6*	10	44369986	T	C	rs41712319
AX-124386523	*FRMD6*	10	44417569	A	G	rs41624350
AX-124381785	*FRMD6*	10	44445350	T	C	rs42444775
AX-106720769	*FRMD6*	10	44481175	A	G	rs42138737
AX-106732937	*FRMD6*	10	44521573	T	G	rs42138402
AX-106737920	*FRMD6*	10	44556318	A	G	-
AX-106763243	*FRMD6*	10	44601357	C	G	rs29026819
AX-115104960	*TNFRSF11B*	14	47438919	T	C	-
AX-106757434	*MAL2*	14	47141282	A	G	rs41255250
AX-115107525	*COLEC10*	14	47267133	A	G	-
AX-169390172	*SAMD12*	14	47826228	A	G	rs110259776
AX-106736159	*SAMD12*	14	47963739	A	G	rs41735513
AX-124375254	*SAMD12*	14	47996787	T	C	rs29024078
AX-106764082	*SAMD12*	14	48021988	A	G	rs41630560
AX-106724218	*SAMD12*	14	48052916	T	C	rs108968111
AX-117082755	*SAMD12*	14	48145874	T	C	rs41603886
AX-185112171	*SAMD12*	14	48191019	A	G	rs110078673

SNP Probe Set ID	Gene symbol	Chr	Position[1]	Allele A	Allele B	SNP rsID
AX-106724034	*CACNA1E/ CAV 2.3*	16	64116934	T	C	rs42663551
AX-124374736	*CACNA1E/ CAV 2.3*	16	64166847	T	G	-
AX-106762254	*CACNA1E/ CAV 2.3*	16	64194978	A	G	-
AX-28511035	*CACNA1E/ CAV 2.3*	16	64246344	A	G	rs109662586
AX-106737399	*CACNA1E/ CAV 2.3*	16	64359255	A	G	-
AX-106752137	*CACNA1E/ CAV 2.3*	16	64387234	T	C	rs110799337
AX-106742186	*ABL2*	16	61960715	A	G	-
AX-117089449	*RYR1*	18	48538054	A	G	-
AX-106742061	*COL1A1*	19	37099312	T	C	rs110819475
AX-106731384	*FBXL7*	20	57301571	A	G	-
AX-124386000	*MEF2A*	21	7145068	A	G	-
AX-124350023	*CNTN4*	22	23365188	A	C	rs110926315
AX-106747756	*CNTN4*	22	23390508	A	G	-
AX-106757157	*CNTN4*	22	23458863	T	C	-
AX-106750212	*CNTN4*	22	23502867	A	C	-
AX-106735713	*CNTN4*	22	23554117	T	C	rs110466322
AX-106742630	*CNTN6*	22	25056533	T	G	rs41583553
AX-124375974	*CNTN6*	22	25076967	T	C	rs110331907
AX-106746728	*CNTN6*	22	25113789	T	G	-
AX-185116096	*CNTN6*	22	25215190	A	G	rs209258798
AX-106753232	*LMOD3*	22	32538832	A	T	rs41642469
AX-106726459	*DST*	23	3468292	T	G	rs109673019
AX-117088037	*DST*	23	3581582	T	C	-
AX-124384326	*RUNX2*	23	18695002	T	C	-
AX-106761391	*RUNX2*	23	18715079	A	G	-
AX-106741590	*RUNX2*	23	18764200	T	C	-
AX-106739513	*CDH7*	24	11204777	T	C	rs110351082
AX-115118013	*THRB*	27	41760090	A	G	rs110994656

[1] Position based on the UMD_3.1.1genome assembly of *Bos taurus*. Chr: chromosome [7].
